# Veterinary Herpesviruses: Experimental Tools for Transcriptomics and Neuroscience

**DOI:** 10.3390/vetsci13030228

**Published:** 2026-02-27

**Authors:** Zsolt Boldogkői, Gábor Torma, Dóra Tombácz

**Affiliations:** Department of Medical Biology, Albert Szent-Györgyi Medical School, University of Szeged, Somogyi u. 4., 6720 Szeged, Hungarytombacz.dora@med.u-szeged.hu (D.T.)

**Keywords:** veterinary herpesvirus, alphaherpesvirus, gammaherpesvirus, transcriptomics, long-read sequencing, nanopore sequencing, RNA isoforms, non-coding RNAs, replication origin, transcriptional interference, transneuronal tract-tracing

## Abstract

Here we discuss three veterinary alphaherpesviruses—pseudorabies virus, equid alphaherpesvirus 1, and bovine alphaherpesvirus 1—that were instrumental in uncovering the true extent of transcriptome complexity through long-read RNA sequencing, which earlier short-read approaches could not resolve. We focus on three major transcriptomic features whose discovery and characterization relied heavily on these viral models: (i) widespread transcriptional overlaps that complicate read assignment and may drive transcriptional interference; (ii) diverse transcript isoforms arising from alternative 5′ and 3′ transcript termini, as well as splicing; and (iii) non-coding RNAs clustered near replication origins that illuminate potential replication–transcription interactions on a shared nuclear template. Long-read viromics in these veterinary systems has additionally served as a stringent benchmark for transcript callers and annotation pipelines, because the extreme density of overlaps and co-terminal transcript families exposes errors that often go unnoticed in typical mammalian transcriptomes. This has made veterinary herpesvirus datasets disproportionately influential in shaping best practices for full-length isoform calling, transcript end mapping, and artifact-robust cDNA library handling. We also discuss animal gammaherpesviruses as proxies for human gammaherpesviruses, allowing experimental investigation of viral programs difficult to study in human infection. Finally, we describe pseudorabies virus applications as a retrograde transneuronal tracer.

## Simple Summary

This review provides an overview of how veterinary herpesviruses—pseudorabies virus of pigs, equine herpesvirus of horses, bovine herpesvirus of cattle, and caviid gammaherpesvirus of guinea pigs—have become powerful research models that advanced two major areas of science. Using a technology called long-read sequencing, which can read complete gene messages from start to stop, scientists discovered that these messages overlap with each other far more extensively than previously known, and that each gene produces many more message variants than expected. These discoveries revealed fundamental principles of how genes are controlled—principles that extend beyond viruses to eukaryotic genomes, including our own. They can also benefit both human and veterinary medicine by providing insights applicable to a broad range of pathogens. Additionally, pseudorabies virus has been repurposed as an essential neurological research tool. Its ability to travel between connected nerve cells makes it uniquely suited for mapping brain circuits, helping scientists visualize brain connectivity with unprecedented precision. Importantly, the virus can also be engineered to carry activity sensors into the brain, allowing direct recording of not only the physical connections between neurons but also their electrical activity—enabling simultaneous study of both the anatomy and the function of neural networks.

## 1. Introduction

Herpesviruses are large double-stranded DNA viruses with a shared virion architecture and a replication cycle. Among veterinary alphaherpesviruses, pseudorabies virus (PRV) causes Aujeszky’s disease in swine, characterized by respiratory illness, neurological signs, and high mortality in young piglets [1]. Equid alphaherpesvirus 1 (EHV-1) is a major pathogen of horses, causing respiratory disease, abortion, and neurological disorders that result in significant economic losses worldwide [2]. Bovine alphaherpesvirus 1 (BoHV-1) is responsible for infectious bovine rhinotracheitis, leading to respiratory and reproductive disorders in cattle [3]. In contrast, caviid gammaherpesvirus 1 (CaGHV-1) represents the gammaherpesvirus subfamily and serves as a promising small-animal model for cross-species studies of this viral group [4]. Together, these four veterinary herpesviruses have provided accessible experimental systems that advanced two major scientific domains.

First, through long-read RNA sequencing (lrRNA-seq), these viruses have revealed previously unrecognized layers of transcriptional complexity—including extensive overlapping transcription, massive isoform diversity, and replication origin-associated RNAs (raRNAs)—whose functional significance remains to be established, but which have already transformed our understanding of viral and, more broadly, eukaryotic genome organization. Second, PRV has become an essential tool for mapping neural circuits, enabling visualization of brain connectivity through transneuronal tracing. These discoveries have broader implications for understanding general transcriptional regulation across eukaryotic systems and the architecture and operation of mammalian neural networks.

**Figure 1:**
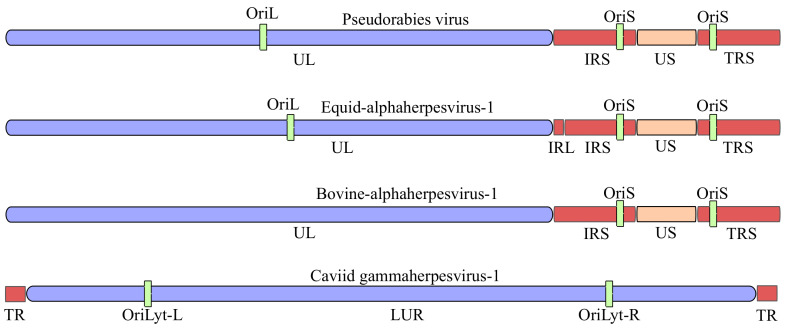
Genome architecture of four herpesviruses. Canonical genome architectures of three alphaherpesviruses pseudorabies virus (PRV), equid alphaherpesvirus 1 (EHV-1) and bovine alphaherpesvirus 1 (BoHV-1), and of the gammaherpesvirus caviid gammaherpesvirus 1 (CaGHV-1). For PRV/EHV-1/BoHV-1, the genomes comprise a unique long (UL) and unique short (US) region flanked by inverted repeat elements of the US region (IRS: internal repeat; TRS: terminal repeat); origins of DNA replication in the US (OriS) and the UL (OriL) regions. For CaGHV-1, terminal repeats (TR) flanking the long unique region (LUR) are shown together with the lytic replication origins (OriLyt-L and OriLyt-R). Genome schematics are not to scale. Abbreviations: UL, unique long region; US, unique short region; IRS/TRS, internal/terminal inverted repeats flanking US; TR, terminal repeat; LUR, long unique region; OriL, origin of replication in the UL region; OriS, origin of replication in the US region; OriLyt-L/R, left/right lytic origin of replication.

These model viruses span two evolutionarily distant herpesvirus subfamilies—Alphaherpesvirinae (PRV, EHV-1 and BoHV-1) and Gammaherpesvirinae (CaGHV-1)—and illustrate subfamily specific genome architectures and compositional biases. The canonical genome organizations and replication origin (Ori) landmarks are summarized in Figure 1, while key comparative genomic features (genome size, GC content and predicted ORF numbers) are compiled in Table 1.

**Figure 2:**
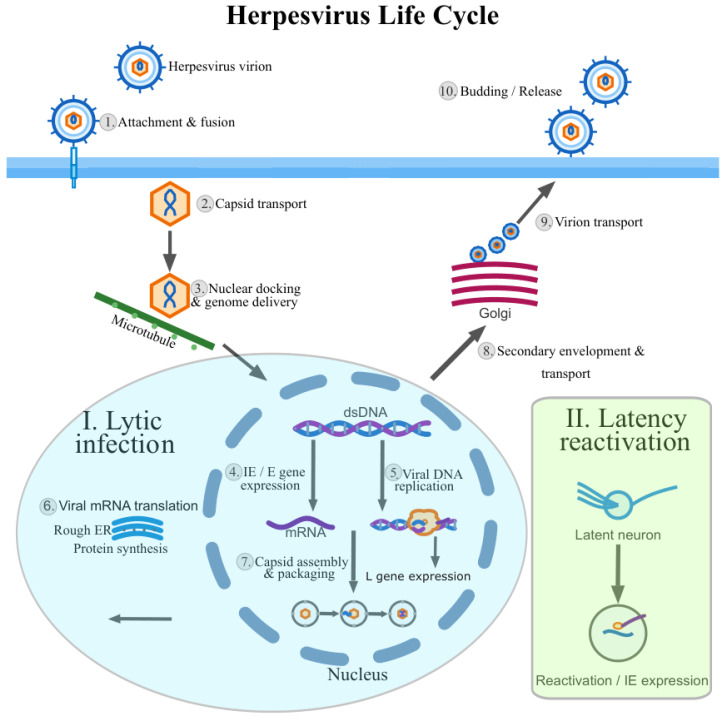
Overview of the herpesvirus life cycle. Herpesvirus entry is initiated by attachment to cellular receptors and membrane fusion (1), followed by cytoplasmic capsid transport along microtubules (2). Capsids dock at the nuclear pore complex and deliver the viral double-stranded DNA genome into the nucleus (3). Productive infection is initiated by immediate-early (IE) and early (E) gene expression (4), which is followed by viral DNA replication (5). Viral proteins are synthesized at the late (L) phase in the cytoplasm, including envelope proteins translated at the rough endoplasmic reticulum (6). Nuclear capsid assembly and genome packaging occur in the nucleus (7). Nucleocapsids subsequently undergo secondary envelopment at Golgi and trans-Golgi network–derived membranes and are transported through the secretory pathway (8–9), culminating in virion release from the cell surface (10). In parallel, a simplified latency–reactivation pathway is shown as an inset. During latency, the viral genome persists in the nucleus as an episomal DNA molecule in specific reservoir cells (illustrated here by a latent neuron). Reactivation leads to the re-initiation of IE gene expression and entry into the productive (lytic) replication cycle.

Following entry, their genomes are delivered to the nucleus, where viral gene expression is driven largely by host RNA polymerase II and associated processing pathways, and is coordinated with viral DNA replication to produce progeny virions [16,17,18]. Their genomes are large by viral standards yet highly compact and transcriptionally crowded. Herpesvirus genes are frequently arranged in tandem clusters and are transcribed from both DNA strands, generating pervasive transcriptional overlaps (TOs). This genome architecture produces large 3′-co-terminal transcripts that share common polyadenylation sites, widespread antisense RNAs, and multigenic transcripts, together yielding a transcriptomic landscape of remarkable structural complexity. Herpesvirus gene expression is temporally regulated. The classical immediate-early (IE), early (E) and late (L) cascade provides a useful conceptual framework, yet pervasive read-through transcription, alternative termination and time-dependent promoter usage frequently blur these categories. Recent studies have further revealed that IE genes initially repress viral transcription before subsequent activation, and that temporal regulation involves complex multi-modular control rather than simple sequential cascades [19,20]. A schematic overview of the herpesvirus life cycle, including lytic replication, latency and reactivation, is shown in Figure 2.

Therefore, transcript architectures differ substantially across infection stages, making time-resolved sampling essential. Latency and reactivation—hallmark features of herpesvirus biology—involve highly restricted transcriptional programs dominated by non-coding RNAs (ncRNAs) that are often low-abundance, cell-type-specific or spatially confined. These ncRNAs regulate viral persistence, immune evasion and reactivation rather than serving as mere transcriptional by-products [21]. Given the pervasive TOs in herpesvirus genomes, transcription complexes are expected to collide frequently as they traverse shared genomic regions. Viral DNA replication and transcription occur simultaneously on the same nuclear template, creating opportunities for direct mechanistic interactions between the replication and transcription machineries. Notably, Oris have a special regulatory role, as they are key control points for initiating and modulating viral DNA synthesis. Herpesviruses thus offer experimentally accessible systems for studying fundamental principles of genome regulation that extend beyond virology [22].

## 2. Sequencing Technologies and Analytical Framework

Short-read RNA sequencing (srRNA-seq) has long been the backbone of transcriptomic studies, computationally assembling transcript structures from fragmented reads while delivering robust quantification and high throughput [23]. This computational assembly, however, faces inherent ambiguity in herpesvirus transcriptomes characterized by extensive TO and polygenic transcription. Alternative transcription start sites (TSSs) and transcription end sites (TESs), antisense RNAs and other ncRNAs are often poorly resolved. The lrRNA-seq technique has addressed these limitations by enabling capture of full-length RNA molecules [24]. Implemented mainly on Pacific Biosciences (PacBio) and Oxford Nanopore Technologies (ONT) platforms, lrRNA-seq allows direct experimental identification of transcript boundaries, isoform structures and multigenic RNAs [25,26]. The core technical characteristics of the sequencing platforms discussed in this section are summarized in Table 2.

ONT direct RNA sequencing (dRNA-seq) uniquely reads native RNA molecules without reverse transcription or amplification [26]. In addition to avoiding cDNA-seq-associated artifacts, dRNA-seq enables detection of epitranscriptomic modifications such as N6-methyladenosine (m6A) [38], 5-methylcytosine (m5C) [39] and pseudouridine [40] via characteristic nanopore current disruptions, and allows inference of adenosine-to-inosine (A-to-I) RNA editing from systematic basecalling discrepancies [32,41]. Its limitations include a requirement for polyadenylated transcripts and coverage biases toward intact RNA molecules [28]. PacBio-based long-read approaches, which typically sequence cDNA, provide highly accurate consensus transcript sequences, but RNA base modifications are generally not preserved through cDNA synthesis; however, SMRT assays that monitor reverse-transcription kinetics can indirectly report certain RNA modifications and structure-dependent RT behavior [31].

A recurring lesson from veterinary herpesvirus datasets is that “platform choice” cannot be separated from “question choice”. If the goal is isoform discovery, transcript boundary mapping, and detection of polygenic RNAs, full-length reads are indispensable. If the goal is fine-grained differential expression with high statistical power across many conditions, short reads remain useful—provided the annotation is already trustworthy. Notably, recent advances in lrRNA-seq technology now enable high-coverage quantification, increasingly blurring this traditional division. In practice, the highest-confidence herpesvirus transcriptomes have been built by using lrRNA-seq to define isoforms and transcript ends. Another practical consideration is library bias. Poly(A)-selection enriches for canonical mRNAs, but can under-represent non-polyadenylated species and some immature or decay intermediates. Conversely, total RNA approaches increase breadth but introduce rRNA-dominated backgrounds and may complicate accurate end calling. For herpesviruses, where closely spaced TESs and frequent read-through transcription are defining features, end-precision—often improved by cap-based or end-enrichment protocols—can matter as much as total depth. Finally, artifact awareness is essential. Template switching, internal priming, and reverse-transcription drop-off can create “ghost isoforms” that appear as alternative polyadenylation or splicing events. Veterinary herpesviruses have been instrumental in identifying these pitfalls because dense co-terminal transcript families provide many opportunities for false-positive isoforms unless filtering rules are explicit and validated across platforms. Common protocol-specific biases and artifacts—particularly critical in overlap-dense herpesvirus transcriptomes—are summarized in Table 3.

Because cDNA-seq-based methods may introduce artifacts such as template switching, current best practice often relies on hybrid strategies combining long-read sequencing (LRS) for structural resolution with short-read sequencing (SRS) for deep quantitative support, alongside orthogonal validation methods such as cap analysis of gene expression (CAGE) [45]. Bioinformatically, Minimap2 is widely used for long-read alignment [46], FLAIR [43], Bambu [47] supports isoform discovery, NAGATA provides nanopore direct RNA–seq-guided transcriptome annotation (particularly useful for compact, gene-dense viral genomes) [48] and the LoRTIA pipeline provides integrated transcriptome annotation [49]. For viral genome assembly from long reads, tools such as Flye [50] enable de novo reconstruction, and in virome/metagenome settings viralFlye extends this workflow by assembling viral contigs from ONT/PacBio long reads and optionally using short reads for polishing when available. Finally, ONT’s Dorado basecaller (RNA004) includes pre-trained models that enable de novo calling of selected RNA modifications (e.g., pseudouridine/Ψ, m6A, inosine and m5C) during direct RNA basecalling [51,52]. Comprehensive reviews of lrRNA-seq methodologies provide detailed protocols for library preparation, platform selection, and analysis workflows across diverse biological systems [53,54,55].

While lrRNA-seq has revolutionized viral transcriptomics, several interpretational challenges warrant careful consideration. ONT dRNA-seq exhibits sequence-context-dependent basecalling errors—reflecting imprecise ionic current readings that result in incorrect base detection—with particularly elevated indel noise in homopolymer-rich regions; however, this is rarely a limiting concern when reads are mapped to a well-annotated reference genome. Additionally, end-related artifacts—including 3′-end instability, frequent 5′ truncation, and soft-clipping or misalignment at both ends—can shift apparent transcript boundaries, yielding spurious TSS/TES calls and misleading isoform models without appropriate filtering [28,37,48]. Quantitative accuracy remains a persistent challenge: long-read methods exhibit non-uniform coverage correlated with transcript length, secondary structure, and modification status, and current normalization strategies often require orthogonal validation. A critical interpretational question concerns low-abundance isoforms, which computational pipelines may detect at <1% of locus expression—these could represent genuine regulatory variants, degradation intermediates, or technical noise, a distinction that remains difficult to resolve without targeted experimental validation. Beyond platform-specific artifacts, a further unresolved issue concerns the risk of biological over-interpretation. Long-read technologies can detect extremely low-abundance transcript variants whose reproducibility across biological replicates is often uncertain. Without orthogonal validation, there is a risk that technical artifacts or stochastic transcriptional events may be annotated as biologically meaningful isoforms. Establishing criteria for functional relevance and reproducibility therefore, remains an important methodological priority in herpesvirus transcriptomics. Finally, because native RNA modifications such as m6A and pseudouridine can alter nanopore signal kinetics and introduce systematic basecalling and alignment deviations, modification-driven signatures may systematically influence transcript model inference. Separating genuine isoform structure from modification-associated signal effects, therefore, requires stringent filtering and orthogonal validation [38,40,48].

## 3. Massive Transcriptional Overlaps

**Figure 3:**
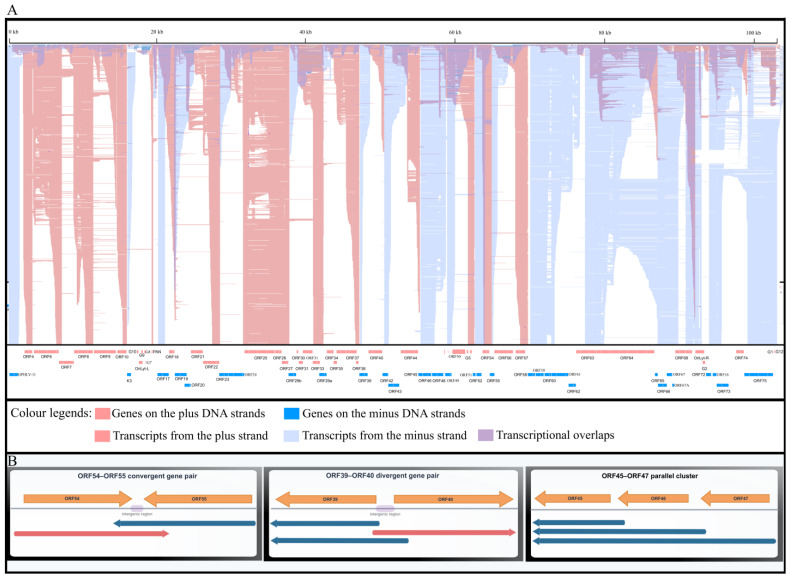
Genome-wide transcriptional overlaps across the caviid gammaherpesvirus 1 (CaGHV-1) genome. (**A**) Strand-specific long-read RNA-seq read alignments displayed in IGV illustrate transcription from the forward (red) and reverse (blue) strands along the complete viral genome. Overlay of signals from the two strands (color blending/shading) highlights pervasive convergent, divergent, and parallel transcriptional overlaps that extend across coding loci and intergenic intervals. (**B**) Representative locus-level schematics illustrating the three major overlap configurations detected by long-read transcript mapping: a convergent overlap at the ORF54–ORF55 locus, a divergent overlap at the ORF39–ORF40 locus (genes separated by ~105 nt), and a parallel/tandem overlap pattern within the ORF45–ORF46–ORF47 gene cluster. These examples illustrate how overlap geometry may promote transcriptional interference (e.g., polymerase collisions in convergent pairs), promoter-level coupling/competition in divergent pairs, and extensive read-through or nested isoforms in tandem gene clusters.

One of the most striking features of herpesvirus transcriptomes is the extent of TOs, where transcripts frequently overlap in convergent (head-to-head, with 3′ ends overlapping), divergent (tail-to-tail, with 5′ ends overlapping), or parallel (same-strand, nested or extending beyond each other) orientations (Figure 3A). Representative locus-level examples of convergent, divergent and parallel overlaps are shown in Figure 3B. Such pervasive TO complicates transcript annotation and quantification, particularly for srRNA-seq, where individual reads may map equally well to multiple transcripts. Importantly, TOs may not be merely biological noise or technical nuisances but could carry genuine biological significance. Antisense transcription can give rise to transcriptional interference [56], altered termination efficiency and polymerase collisions [22]. lrRNA-seq studies in veterinary alphaherpesviruses—most notably PRV [57] revealed that extensive TO is a defining organizational principle rather than a rare exception. These analyses uncovered dense networks of overlapping coding and non-coding transcripts that were largely invisible to srRNA-seq-based approaches [58]. Similar conclusions emerged from comprehensive EHV-1 [59] and BoHV-1 [14] transcriptome maps, underscoring that TO is a conserved and likely functional feature of herpesvirus genome organization [60].

In PRV, lrRNA-seq has revealed that TO patterns extend well beyond the long-known parallel TOs within tandem gene clusters: divergent gene pairs can generate extensive head-to-head overlaps through very long alternative 5′-untranslated regions (UTRs), while convergent loci frequently produce readthrough RNAs that embed antisense segments relative to the partner gene [61]. Two major types of convergent TO can be distinguished: “hard” overlaps occur when the canonical transcripts of both genes intrinsically overlap—that is, the TES of one canonical transcript lies within the neighboring convergent gene—whereas “soft” TOs arise from occasional readthrough events extending into the partner gene. Although convergent genes are often separated by relatively long, commonly repetitive intergenic regions, “soft” TOs via readthrough occur in essentially every convergent gene pair, while “hard” TOs (e.g., PRV ul7/8, ul30/31, ul50/51 gene pairs) remain rare exceptions [61].

EHV-1 provides an even more overlap-dense example: nearly every divergent gene pair produces transcripts with extensive head-to-head overlap, and the transcriptome contains very long overlaps spanning multiple genes. Canonical convergent transcripts usually show only “soft” TOs via occasional readthrough, but the ORF29/30 (ortholog of PRV ul31/30) pair represents a rare “hard” TOs exception [59]. In BoHV-1, time-course long-read analyses further revealed the complexity of this overlapping meshwork organization [60].

### Biological Implications of Transcriptional Overlaps

The mechanistic implications of pervasive TO extend beyond annotation complexity. When RNA polymerase II complexes transcribe convergent genes simultaneously, they are expected to collide as they approach their respective polyadenylation sites. Studies in Saccharomyces cerevisiae demonstrated that convergent gene pairs exhibit transcriptional interference mediated by 3′ UTRs, where readthrough transcription from one gene suppresses expression of its convergent partner [56]. Whether similar interference operates in herpesvirus genomes remains experimentally unexplored, but the conservation of convergent gene arrangements across herpesvirus subfamilies suggests either tolerance of interference or active exploitation for regulatory purposes [22]. Beyond polymerase collisions, overlapping transcription may also regulate gene expression through RNA duplex formation or competition for shared transcription factors—mechanisms that could enable rapid, coordinated temporal control during the lytic cycle without requiring dedicated regulatory proteins. Studies of human cytomegalovirus (HCMV) have shown that divergent promoter pairs can drive coordinated expression through shared transcription factor binding sites, and that replication-associated long non-coding RNAs actively regulate viral DNA synthesis through formation of RNA:DNA hybrids [62]. The extraordinarily long 5′ UTR isoforms observed in divergent gene pairs likely reflect active transcription through intergenic regulatory regions, suggesting that apparent overlap may represent functional scanning of regulatory landscapes. Understanding how widespread antisense transcription functions in herpesvirus biology will require integration of long-read transcriptome data with functional genetic studies and single-cell approaches. While pervasive transcriptional overlaps are robustly documented across multiple alphaherpesviruses, their functional interpretation remains debated. Several models propose active transcriptional interference, polymerase collision, or regulatory coupling between convergent gene pairs as biologically meaningful mechanisms. In contrast, alternative interpretations suggest that a substantial fraction of overlaps may reflect permissive transcription within compact viral genomes, or represent transcriptional noise arising from high promoter density and extensive readthrough activity, without specific adaptive regulatory function. Distinguishing genuine regulatory interactions from architectural consequences of genome density or stochastic transcription remains a key unresolved challenge in herpesvirus transcriptomics.

## 4. Transcript Isoforms: Splice Variants and 5′/3′ Termini Diversity

A second major layer of herpesvirus transcriptome complexity arises from transcript isoform diversity. Individual genomic loci often give rise to multiple transcripts initiated from distinct TSSs, terminated at alternative TESs, and in some cases processed by splicing. Although the prevalence of splicing varies among herpesvirus subfamilies, alternative transcript boundaries and nested transcript organization are nearly universal. Alternative TSS usage can reflect multiple promoters within a locus, temporal shifts in promoter activity during infection, or processing of read-through transcripts. Herpesviruses exhibit substantially greater TSS variability than TES variability—unlike poxviruses [63], for example, where the opposite pattern is observed. TES variability and alternative polyadenylation generate co-terminal transcript families with different 3′ untranslated region lengths, potentially influencing RNA stability, localization and post-transcriptional regulation. While srRNA-seq can detect splice junctions, it faces two fundamental limitations in isoform reconstruction: (1) it cannot unambiguously assign spliced reads to specific TSS–TES combinations, and (2) when transcripts contain two or more introns, it cannot reliably determine which introns co-occur within the same mature transcript molecule. lrRNA-seq overcomes this limitation by capturing complete transcript structures in single reads.

PRV illustrates the scale of transcript-termini diversity particularly clearly: the updated atlas reported numerous TSS isoforms with variable 5′-UTR lengths and fewer TES variants altering 3′-UTR boundaries under stringent filtering [61]. A particularly informative observation is that some long TES isoforms traverse the intergenic region (e.g., UL27-AT, UL35-AT, UL44-AT, CTO-S-AT, US2-AT), potentially impacting downstream gene regulation or creating novel TOs. Crucially, PRV also revealed a large class of 5′-truncated RNAs that are 3′-co-terminal with canonical mRNAs: many of these initiate within ORFs, carry in-frame ATGs, and share the canonical stop codon—consistent with the potential for N-terminally truncated polypeptides—whereas the remainder are likely non-coding [61]. This nested/co-terminal architecture blurs the boundary between “TSS isoforms” and distinct coding units and directly motivates LRS-based full-length validation when interpreting intragenic initiation [61]. Replication-origin loci further highlight how TES/TSS heterogeneity interacts with regulatory hubs: CTO-S is extremely abundant in PRV, and the Ori-associated region contains additional low-copy isoforms and antisense transcripts, including CTO-as and UL21-as, alongside novel CTO-S variants (e.g., a novel TES isoform CTO-S-AT2 and CTO-S-cx RNA) [61].

BoHV-1 time-course long-read profiling adds two concrete isoform principles that are hard to recover with SRS alone. First, a single promoter can generate multiple TSSs with gene-specific distribution patterns that remain reproducible across timepoints, providing a quantitative handle on promoter “micro-heterogeneity” [60]. Second, the bicp4 locus shows unusually rich transcript-end diversity. TSS isoforms initiate from both the 5′-UTR and within the ORF, while TES isoforms—rare for herpesvirus genes—terminate in both the 3′-UTR and within the ORF. The locus also generates 3′-truncated ncRNAs and ncRNAs overlapping long UTR isoforms [60]. In parallel, bicp22 exhibits one of the most complex architectures, combining TSS polymorphism, multiple splice patterns, very long readthroughs spanning much of the US region, and detectable antisense transcription; its short versus long TSS isoforms also differ in upstream ORF (uORF) content, providing a plausible route for translational modulation layered onto transcript-structure diversity [60]. EHV-1 extends isoform complexity into both splicing and termination logic. The hybrid atlas reported abundant fusion/chimeric transcripts, some compatible with chimeric proteins. Notably, many upstream genes within tandem clusters possess their own TESs in addition to shared co-terminal ends—an organization far less prominent in related alphaherpesviruses [59]. EHV-1 encodes a CTO-S homolog near OriL and detects CTO-L as a TES isoform of the ul21 homolog (ORF40) co-terminal with canonical CTO-S.

### Functional Implications of Isoform Diversity

The massive isoform diversity revealed by lrRNA-seq raises fundamental questions about regulatory complexity and functional specialization. While some isoforms clearly encode distinct protein products with different functional domains, the biological roles of many TSS and TES variants remain speculative. Alternative 5′ UTRs may regulate translation efficiency or subcellular localization, while 3′ UTR variants could influence mRNA stability or microRNA-mediated regulation. However, for the majority of identified isoforms, functional validation through targeted mutagenesis or isoform-specific knockdown remains lacking. Distinguishing functionally relevant isoforms from transcriptional noise represents a critical challenge for the field.

## 5. Replication Origin-Associated RNAs: CTO and NOIR Families

**Figure 4:**
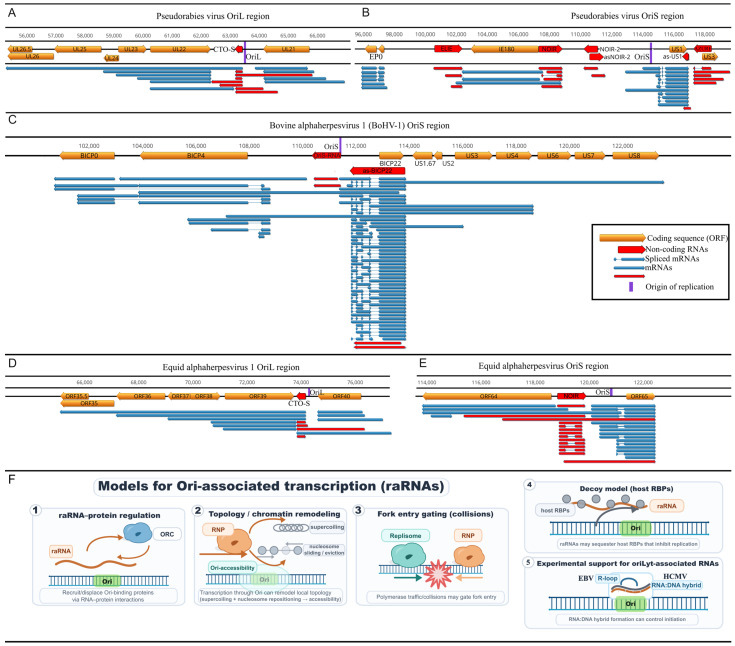
Transcriptional architectures surrounding lytic replication origins (OriL/OriS) in varicelloviruses. Panels show (**A**) pseudorabies virus (PRV) OriL, (**B**) PRV OriS, (**C**) bovine alphaherpesvirus 1.1 (BoHV-1) OriS, (**D**) equid alphaherpesvirus 1 (EHV-1) OriL, and (**E**) EHV-1 OriS. Protein-coding genes (orange), origin-associated RNAs (red), and origin positions (purple) are shown together with long-read-mapped transcript isoforms (blue). The comparison highlights that Ori loci are embedded in dense, frequently overlapping transcription, with multiple long isoforms traversing or flanking the origins. (**F**) Conceptual models for how Ori-associated transcription/raRNAs may influence replication initiation: (1) raRNA–protein interactions that recruit or displace Ori-binding factors, (2) transcription-through-Ori driven changes in local DNA topology and chromatin accessibility (supercoiling and nucleosome repositioning), (3) polymerase traffic and collisions that gate replication fork entry, and (4) a decoy mechanism in which raRNAs titrate host RNA-binding proteins. Functional evidence for RNA:DNA hybrid (R-loop) formation at oriLyt and its role in replication initiation is indicated from representative gammaherpesvirus studies.

A particularly distinctive contribution of veterinary herpesvirus models concerns transcription around the Oris (Figure 4). lrRNA-seq studies revealed that Ori regions are frequently embedded within dense transcriptional landscapes enriched for ncRNAs. In alphaherpesviruses, several classes of replication origin-associated RNAs have been described, including transcript families referred to as CTO (*Close to the OriL*) transcripts, which are long polygenic RNAs associated with OriL, and NOIR (*Non-coding RNA in the Inverted Repeat*), which are transcribed from regions flanking OriS, respectively [22,64]. CTO transcripts have been identified in PRV and EHV-1 but are absent in BoHV-1, which lacks OriL. In EHV-1, the longer CTO-S isoform’s TATA box is co-localized with OriL [59]. In contrast, NOIR-like transcriptional activity at OriS appears to be a conserved feature of varicelloviruses: NOIR homologs or functionally analogous transcripts have been detected in all varicellovirus genomes subjected to comprehensive LRS. For example, in varicella-zoster virus and simian varicella virus, NOIR-like RNAs are present, while in BoHV-1, which lacks a canonical NOIR gene, a very long 5′-UTR isoform of the us1 homologue may fulfill this function [64]. In PRV, the OriS region harbors not only NOIR-1 but also NOIR-2, which is transcribed convergently to NOIR-1. In BoHV-1, OriS-RNA may mediate similar NOIR-2-like activity [14]. These ncRNAs form structured transcriptional environments around the Ori, which may influence replication initiation, local chromatin organization, or replication–transcription interference.

In alphaherpesviruses, OriS is flanked by the two master transcriptional regulators—icp4 and us1—with extended TSS isoforms that span the Ori itself. In BoHV-1, the OriS region harbors an exceptionally dense regulatory architecture, including long, oppositely oriented IE transcripts overlapping OriS: long TSS isoforms of bICP4 and bICP22. Notably, the bICP22 promoter/TSS region itself overlaps OriS, reinforcing the idea that replication-initiation and local transcription initiation can physically and functionally intersect [60]. These long isoforms overlap both OriS and each other, creating a multi-layered transcriptional architecture. This genomic configuration suggests that the OriS region functions as a super-regulatory hub where DNA replication and global transcriptional control are spatially integrated and likely subject to reciprocal interference. These observations have broader implications for understanding how replication and transcription are coordinated on compact eukaryotic DNA genomes.

A key open question is whether Ori-associated transcription actively triggers replication initiation or merely reflects the high regulatory activity concentrated at these genomic hubs. Several non-mutually exclusive mechanisms have been proposed based on existing transcript maps [64]. First, raRNAs may directly regulate Ori function by recruiting or displacing Ori-binding proteins through RNA–protein interactions. Second, the act of transcription through Ori may mechanically alter local chromatin structure—changing supercoiling and nucleosome positioning—thereby modulating Ori accessibility. Third, transcriptional polymerase traffic may create regulated collisions that control when and where replication forks can enter. Fourth, raRNAs may act as molecular decoys, sequestering host RNA-binding proteins that would otherwise inhibit viral replication. Supporting a direct regulatory role, functional studies in betaherpesviruses [62] and gammaherpesviruses [65] have demonstrated that replication-associated RNAs control DNA synthesis initiation through formation of RNA:DNA hybrids. Veterinary herpesviruses provide ideal systems for testing these models experimentally, as Ori regions and their flanking promoters can be genetically manipulated and the consequences measured through replication kinetics and transcriptome profiling. Importantly, these models are not mutually exclusive and may operate in different temporal windows of lytic infection; this mechanistic ambiguity motivates targeted perturbation experiments at Ori-flanking promoters (summarized in Figure 4F).

Another underappreciated implication is that Ori-regions are hotspots for annotation artifacts unless end calling is precise. Because multiple long isoforms can traverse Ori regions from both directions, incomplete cDNAs may appear as distinct short ncRNAs. Direct RNA sequencing and cross-platform confirmation in PRV/EHV-1/BoHV-1 have therefore been critical not only for the precise characterization of CTO/NOIR isoforms but also for establishing stringent criteria for calling authentic raRNAs.

### Open Questions Regarding Origin-Associated RNAs

While CTO and NOIR transcripts represent reproducible features of alphaherpesvirus transcriptomes across multiple platforms and viral species, their precise functions remain largely speculative and direct mechanistic evidence is limited. Whether these RNAs act primarily in cis (affecting the local origin) or in trans (regulating distant genomic regions) remains unresolved. Experimental approaches such as CTO/NOIR depletion followed by replication kinetics assays will be essential to validate proposed functions and distinguish regulatory roles from incidental transcriptional byproducts.

## 6. Caviid Gammaherpesvirus-1 as a Gammaherpesvirus Model

Veterinary herpesviruses contribute to our understanding of viral transcriptomics not only through alphaherpesviruses but also via gammaherpesvirus models. Human gammaherpesviruses, such as Epstein–Barr virus (EBV) [66,67] and Kaposi’s sarcoma-associated herpesvirus (KSHV) [68,69], are major pathogens associated with lymphoproliferative diseases and malignancies. However, ethical and technical constraints limit experimental manipulation in human subjects.

Animal gammaherpesviruses, such as murine gammaherpesvirus 68 (MHV68) [70,71], overcome these barriers through controlled infection and genetic manipulation. However, MHV68 has important limitations: limited sequence homology to human gammaherpesviruses, lack of key regulatory elements found in KSHV and EBV, and significant physiological differences in latency establishment.

Caviid gammaherpesvirus-1 (CaGHV-1), originally identified in 1969 [72] and recently reclassified as a rhadinovirus [4], exhibits remarkable genomic and functional similarity to KSHV. The virus encodes 75 predicted ORFs, including orthologues of key KSHV oncogenes: ORF73 (LANA), ORF50 (RTA), and the PAN ncRNA essential for lytic replication in KSHV but absent in MHV68. Torma and colleagues (2025) [15] conducted the first comprehensive lrRNA-seq analysis of CaGHV-1, revealing extensive transcript complexity mirroring KSHV. The study identified monogenic mRNAs, polygenic transcripts, complex transcripts, and antisense RNAs. Using LoRTIA [49], TSSs were mapped at single-nucleotide resolution, identifying TATA boxes and the TATTWAA motif essential for late gene transcription in KSHV, EBV, and HCMV. Splicing patterns in key regulatory genes showed remarkable conservation with KSHV: CaGHV-1 ORF50 (RTA) contains four exons matching KSHV ORF50, and ORF57 splicing mirrors its KSHV orthologue. Additionally, extensive transcriptional complexity was identified in the G4–G5 region and ORF63–64 locus, with multiple splice variants paralleling KSHV [69]. Furthermore, genome-wide TOs were revealed between convergent, divergent, and co-oriented genes. “Hard” TOs were identified in convergent clusters (ORF18–ORF19, ORF64–ORF65, ORF74–ORF75), and “soft” TOs in others (ORF10-K3, G4-ORF52)—patterns strikingly similar to EBV [67] and KSHV [69]. Numerous raRNAs were detected near both lytic Oris, including transcripts overlapping origins and long complex RNAs encompassing Oris, phenomena documented in KSHV, EBV, and HCMV [64]. These findings suggest that the transcriptomic principles uncovered through veterinary herpesvirus models have broad applicability across the Herpesviridae family.

Beyond transcriptome architecture, CaGHV-1 offers experimental advantages for studying gammaherpesvirus latency and reactivation dynamics. Recent comprehensive analysis of the EBV- transcriptome using long-read sequencing revealed extensive diversity in lytic gene isoforms and identified biphasic promoters with features of both early and late regulation, demonstrating the complexity of temporal control in gammaherpesviruses [73]. The oncogenic potential of CaGHV-1 in guinea pigs additionally provides a physiologically relevant model for studying gammaherpesvirus-driven lymphoproliferation. Unlike MHV68, which requires specific genetic backgrounds to induce lymphoma, CaGHV-1 spontaneously drives B cell proliferative disease in immunocompetent guinea pigs, more closely recapitulating the pathogenesis of KSHV-associated malignancies [74]. Integration of long-read transcriptome analysis with tumor genomics in this system could illuminate viral transcriptional programs that promote transformation.

The guinea pig model also offers practical advantages over non-human primate models: lower cost, established husbandry, and fewer ethical constraints, while maintaining closer biological similarity to human gammaherpesviruses than MHV68 [4].

## 7. Pseudorabies Virus as a Transneuronal Tracer

Attenuated and genetically engineered PRV strains are widely used as retrograde transneuronal tracers for mapping neural circuits, owing to their ability to infect neurons, replicate efficiently, and spread across synaptically connected networks in a directionally controlled manner [75,76]. Several biological properties make PRV particularly well suited for this application. First, PRV exhibits robust neurotropism and efficient axonal transport, allowing it to traverse multisynaptic pathways with high fidelity. Second, the temporal progression of infection can be experimentally controlled using replication-competent, attenuated or replication-deficient strains, enabling time-resolved dissection of neuronal connectivity. Third, PRV tolerates substantial genome engineering, permitting insertion of reporter genes without compromising viral spread. Recent comprehensive protocol reviews provide detailed methodologies for exploiting PRV’s transneuronal capabilities in both peripheral injection paradigms and direct CNS applications [77].

PRV-based circuit tracing has been applied to diverse neural systems, demonstrating the versatility of the approach. The virus has been used to map autonomic pathways controlling peripheral organs such as brown adipose tissue, bone marrow, and the cardiovascular system [78,79]. A landmark example is the PRV-based mapping of brain-bone sympathetic circuits, which revealed that central sympathetic outflow to bone originates from 87 distinct brain nuclei across six brain divisions, with site-specific variation in infection levels suggesting hierarchical organization of bone innervation [79].

Beyond traditional anatomical tracing, recombinant PRV strains have been engineered to carry genetically encoded activity sensors such as ratiometric calcium indicators, enabling optical monitoring of neural activity in virally labeled circuits [80]. These activity sensor PRVs permit researchers to both identify synaptically connected neurons and simultaneously record their functional responses to physiological or pharmacological stimuli. Timer PRVs expressing two fluorescent proteins with different kinetics have been developed to define temporal windows early after infection, allowing functional interrogation during periods when neuronal physiology remains relatively intact. Furthermore, comparative studies using PRV alongside rabies virus strains have demonstrated that while PRV enables robust multisynaptic tracing, rabies virus can provide superior Golgi staining-like visualization of dendritic morphology and spine density in specific neuronal populations [81].

Multicolor rainbow PRVs expressing spectrally distinct fluorescent proteins facilitate the simultaneous tracing and differentiation of multiple parallel circuits within complex brain regions. PRV has also been explored as a gene delivery vector for experimental neuroscience and gene function studies [80]. Compared with non-viral delivery systems, PRV-based vectors provide high transgene expression levels, efficient neuronal infection and the ability to target defined neural circuits. Unlike human herpesviruses, PRV can be used in animal models with fewer biosafety and ethical constraints, while still preserving key biological properties relevant to alphaherpesvirus biology.

The genetic tractability that makes PRV valuable for circuit tracing also presents opportunities for studying alphaherpesvirus biology in physiologically relevant neuronal contexts. While cultured cell systems have yielded fundamental insights into herpesvirus replication and gene expression, they fail to recapitulate the complex cellular environments and restricted transcriptional programs characteristic of neuronal infection. Recent studies have revealed that viral gene expression differs substantially between peripheral neurons and CNS neurons, and between neurons supporting productive replication versus those establishing transient quiescence, suggesting that cell-type-specific host factors influence viral transcriptional programs.

## 8. Future Directions

This review focuses on veterinary alphaherpesviruses for which high-resolution long-read transcriptomic data are available (PRV, EHV-1, BoHV-1, and CaGHV-1), while many other veterinary herpesviruses remain underexplored. Functional validation of individual transcript isoforms is limited, and current bulk sequencing approaches cannot resolve cell-to-cell heterogeneity or spatial organization of viral transcription. Emerging long-read sequencing technologies are poised to address these limitations and further refine our understanding of herpesvirus transcriptional complexity. Integration of nanopore direct RNA sequencing with single-cell approaches will enable resolution of cell-to-cell heterogeneity during viral latency and reactivation, which remains largely inaccessible to bulk analyses [82]. Targeted long-read enrichment strategies, recently applied to hepatitis B virus and JC polyomavirus transcriptomes, allow deep profiling of low-abundance viral transcripts in clinical samples [83,84]. High-accuracy long-read platforms with dual unique molecular identifiers now permit detection of single-nucleotide variants and isoform-specific mutations within viral RNAs, providing new insights into genotype–phenotype relationships [85]. At the single-cell level, long-read sequencing will reveal cell-type-specific transcriptional programs, elucidating how individual neurons differentially regulate viral gene expression during latency and reactivation.

Beyond transcriptomics, PRV-based neural circuit tracing continues to evolve. Inducible neural tracing systems enabling temporal control over viral spread and foreign gene expression represent a promising avenue for enhancing the precision of PRV-based circuit mapping. Application of these emerging technologies to a broader range of veterinary herpesviruses will expand our understanding of transcriptional diversity across the Herpesviridae family.

## 9. Concluding Remarks

Research on veterinary herpesviruses has substantially advanced our understanding of the molecular mechanisms governing herpesvirus gene expression. In recent years, this work—particularly through transcriptomic investigations—has helped shift the field from largely inference-based models toward experimentally validated frameworks, uncovering fundamental principles of viral transcriptional regulation with broad relevance to eukaryotic systems. Long-read sequencing of PRV, EHV-1, BoHV-1, and CaGHV-1 has demonstrated that herpesvirus genes generate far more extensive isoform repertoires than previously recognized, encompassing diverse transcription start sites, polyadenylation sites, and splice variants. These studies have uncovered remarkably long polygenic and complex transcripts, revealing unexpected features of herpesvirus gene architecture. The transcriptional landscape is characterized by massive transcriptional overlaps in convergent, divergent, and parallel orientations, challenging traditional models of discrete transcription units.

Veterinary herpesvirus research has also led to the identification of functionally important transcript classes with broader significance. The OriL-associated non-coding CTO transcripts, identified in PRV and EHV-1, and the OriS-associated NOIR transcript family, which extends to human herpesviruses such as VZV, exemplify origin-proximal non-coding RNAs with potential regulatory functions. Critically, studies on these viruses revealed that extended 5′ and 3′ isoforms of origin-flanking genes frequently overlap or even initiate from within replication origins. In BoHV-1, the two master transcriptional regulators—icp4 and us1—flank OriS with extended TSS isoforms that span the origin itself, and these genes also transcribe into each other. These findings support the hypothesis—though not yet definitively proven—that origin-proximal regions function as ‘super-regulatory hubs’ coordinating transcription and DNA replication. While the structural features of these transcripts are robustly documented across multiple platforms and viral species, direct experimental evidence for their regulatory functions remains limited. Testing this paradigm through targeted deletion or depletion experiments represents a critical priority for the field.

In parallel, animal gammaherpesviruses provide indispensable systems for studying gammaherpesvirus transcription under conditions that are ethically and technically inaccessible in humans. Beyond transcriptomic discoveries, PRV has independently transformed systems neuroscience through its development as a transneuronal circuit tracer. Together, these two trajectories illustrate how veterinary herpesvirus models have simultaneously reshaped both molecular transcriptomics and systems neuroscience, and are likely to remain central to both fields as long-read technologies continue to mature. The regulatory principles uncovered through veterinary herpesvirus transcriptomics—extensive overlapping transcription, multifunctional genomic elements, and origin-proximal regulatory hubs—may represent universal solutions to the challenge of encoding maximal regulatory complexity within limited genomic space. Whether analogous strategies operate in other organisms—including ones with more complex genomes—remains an open question that emerging long-read technologies are well positioned to address.

## Figures and Tables

**Table 1:** Genomic architecture and basic genomic characteristics of four herpesvirus model species. The table summarizes the subfamily assignment, canonical genome organization, genome size, GC content, predicted number of protein-coding open reading frames (ORFs), and replication origin elements for selected veterinary herpesviruses. UL and US denote the unique long and unique short genome regions, respectively. IRS/TRS indicate the internal and terminal inverted repeat elements flanking the US regions, respectively, and enabling genome isomerization. OriL and OriS denote replication origins associated with the long and short genome regions in alphaherpesviruses, whereas OriLyt-L and OriLyt-R indicate the left and right lytic replication origins in gammaherpesviruses. Genome sizes, GC contents and ORF numbers correspond to the respective reference genome assemblies reported in the cited sources.

Virus	Subfamilies	Genome Architecture	Genome Size (bp)	GC (%)	Protein-Coding ORFs	Replication Origins (Ori)
Pseudorabies virus (PRV)	Alphaherpesvirinae [5]	UL and US regions; US flanked by internal and terminal inverted repeats (IR/TR) enabling genome isomerization [6]	143,461 [6]	~74.5 [6]	72 [6]	OriL and two copies of OriS [6]
Equid alphaherpesvirus 1 (EHV-1)	Alphaherpesvirinae [5]	UL and US regions with inverted repeats flanking US; conserved alphaherpesvirus gene order [7]	150,223 [7]	56.7 [8]	80 [9]	OriL and two copies of OriS [9]
Bovine alphaherpesvirus 1 (BoHV-1)	Alphaherpesvirinae [5]	UL and US regions separated and flanked by internal and terminal repeats (IR/TR) [10]	~135,000 [11]	75 [12]	73 [13]	Two copies of OriS; no OriL identified [14]
Caviid gammaherpesvirus (CaGHV-1)	Gammaherpesvirinae [4]	Rhadinovirus-like genome composed of a large unique region and terminal repeat elements [4]	103,374 [4]	35.45 [4]	75 major ORFs [4]	Two lytic origins (OriLyt-L and OriLyt-R); latent origin not identified [15]

**Table 2:** Technical characteristics of sequencing technologies. Short-read RNA-seq (Illumina), PacBio Iso-Seq/HiFi, and ONT direct RNA and cDNA-seq-based protocols are compared in terms of read length/accuracy/throughput, end/isoform resolution, and RNA modification capability. Abbreviations: SRS, short-read sequencing; LRS, long-read sequencing; RT, reverse transcription; TES, transcription end site; TSS, transcription start site; dRNA-seq, direct RNA sequencing.

Technology	Read Length/Accuracy/Throughput	Ends and Isoforms	RNA Modification Capability
Short-read RNA-seq (Illumina)	Short reads (50–300 bp), very high throughput, very high per-base accuracy (Q30 ≈ 99.9%) [27]	Transcript structures and TSS/TES inferred computationally; overlapping herpesvirus transcripts cause ambiguity [22]	Not supported (cDNA-seq-based) [28]
PacBio Iso-Seq/HiFi (cDNA-seq)	Long full-length cDNA-seq reads (often up to >10 kb); HiFi consensus accuracy typically ≳ 99.8% [29,30]	High-confidence splice isoforms and transcript ends when full-length molecules are captured [22]	Generally not preserved (cDNA-seq); only indirect RT-kinetics approaches possible [31]
ONT dRNA-seq	Native long RNA molecules; lower throughput than cDNA; lower raw accuracy but improving with chemistry/base caller [32]	Excellent TES and poly(A)-anchored end detection; direct intron validation without RT artifacts; systematic 5′ truncation (~10–15 nt) and 3′ bias [26,32]	Direct detection from current signal (Ψ, m6A, m5C, inosine via Dorado/RNA004; validation recommended) [28,33]
ONT direct cDNA-seq (PCR-free)	Long reads; typically higher yield than dRNA-seq; raw accuracy model-dependent [34]	Good isoform and splice discovery; end precision depends on RT and priming [35,36]	Not supported (cDNA-seq) [28]
ONT PCR-cDNA-seq	Long reads; usually highest yield among ONT RNA workflows; suitable for multiplexing/low input [34,37]	Isoform discovery is possible but end precision is affected by RT and PCR artifacts [35,36]	Not supported (cDNA-seq) [28]

**Table 3:** Typical biases and artifacts of RNA sequencing protocols and their practical impact in overlap-dense herpesvirus transcriptomes. Major protocol-specific biases (e.g., 3′ bias and 5′ truncation in dRNA-seq; internal priming and RT/PCR artifacts in cDNA-seq-based workflows) are summarized together with their consequences for transcript boundary calling and isoform inference.

Technology	Major Biases/Artifacts	Practical Impact in Herpesvirus Datasets
**Short-read RNA-seq (Illumina)**	Ambiguous read assignment in overlap-dense genomes; incomplete resolution of antisense, polygenic and co-terminal transcripts [22]	Isoform inflation or mis-quantification unless transcript models are trusted
**PacBio Iso-Seq/HiFi (cDNA-seq)**	RT and optional PCR introduce drop-off and chimera artifacts; incomplete 5′ coverage when cDNA synthesis fails [42,43]	Truncated 5′ ends and rare artificial isoforms
**ONT dRNA-seq**	Requires poly(A)+ RNA; strong dependence on RNA integrity; systematic 3′ bias and 5′ truncation (~10–15 nt) [26,44]	TSS mapping is less precise; TES and read-through transcription are highly reliable
**ONT direct cDNA-seq**	RT-associated template/strand switching; internal oligo(dT) priming unless filtered [35,36]	False TES-like ends and spurious isoforms in co-terminal transcript families
**ONT PCR-cDNA-seq**	PCR bias and chimera formation; RT- and priming-related artifacts remain [35,36]	Over-representation of specific isoforms; false transcript structures without artifact-aware filtering

## Data Availability

No new data were created or analyzed in this study. Data sharing is not applicable to this article.
